# Beyond Reading and Understanding: Health Literacy as the Capacity to Act

**DOI:** 10.3390/ijerph15081676

**Published:** 2018-08-07

**Authors:** Jany Rademakers, Monique Heijmans

**Affiliations:** 1 Netherlands Institute for Health Services Research, 3500 BN Utrecht, The Netherlands; m.heijmans@nivel.nl; 2 Department of Family Medicine, School for Public Health and Primary Care (CAPHRI), Maastricht University, 6200 MD Maastricht, The Netherlands

**Keywords:** health literacy, health determinants, health competencies, health outcomes, patient-centered care, definitions, conceptual models

## Abstract

Many health literacy interventions have a limited focus on functional/cognitive skills. In psychosocial models, the capacity to act however is seen as a major driver of behavioural change. This aspect is often lacking in health literacy concepts. In this study, we examine the impact of both aspects of health literacy (functional/cognitive and capacity to act) on specific healthcare outcomes (healthcare use, experiences with patient-centered care, shared-decision making, and self-management). In a sample of a national panel of people with a chronic disease (NPCD), questions about health literacy, patient activation, and outcomes were asked. The results indicated that 39.9% had limited HL levels and 36.9% had a low activation score. Combined, 22.7% of the sample scored low on both aspects, whereas 45.8% had adequate levels on both. Patients who score low on both use more healthcare and have less positive experiences with patient-centered care, shared decision making, and self-management. Patients who have adequate competency levels in both respects have the best outcomes. Both cognitive and non-cognitive aspects of health literacy are important, and they enhance each other. The capacity to act is especially important for the extent to which people feel able to self-manage.

## 1. Introduction

Health literacy is regarded as an important prerequisite in order to take up a pro-active role with respect to one’s health and lifestyle, and to be able to perform as an active partner in encounters with healthcare professionals and institutions. This is especially relevant for people with one or more chronic conditions, who have more frequent interactions with healthcare professionals and who are supposed to be active in the self-management of their disease.

In the past decennia, worldwide many definitions and conceptualizations of health literacy have emerged. At first, health literacy was defined as having basic reading and writing skills (which are essential e.g., to read consent forms, information leaflets and medicine labels). This is referred to as ‘functional health literacy’. A more elaborate and much used model is the one proposed by Nutbeam in 2000 which distinguishes between functional, interactive and critical health literacy [1]. On top of the basic functional skills Nutbeam introduced interactive (also known as ‘communicative’) literacy: more advanced cognitive and literacy skills which, together with social skills, can be used to actively participate in everyday situations, extract health information and derive meaning from different forms of health communication, and to apply new information to changing circumstances. Critical health literacy refers to more advanced cognitive and social skills needed to critically assess health information and to use this information in one’s personal situation [1]. In 2012, the Health Literacy Survey-Europe (HLS-EU) consortium developed a new conceptual model in which 12 dimensions of health literacy were discerned: four competency levels related to accessing, understanding, appraising, and applying health information in the three domains of healthcare, disease prevention, and health promotion [2]. Both the health literacy models of Nutbeam and the HLS-EU consortium address a broader range of competences compared to the original functional definition, such as communication and social skills and the ability to apply health information. Cognitive aspects and information processing, however, remain central elements in the models. A recent review (2017) on definitions and models of health literacy in childhood and youth discerned three different key dimensions of health literacy: (1) cognitive attributes (knowledge, functional health related skills, comprehension and understanding; appraisal and evaluation, critical thinking); (2) behavioural and operational attributes (seeking and accessing information; communication and interaction; application of information; other context specific skills; citizenship); and (3) affective and conative attributes (self-awareness and self-reflection; self-control and self-regulation; self-efficacy; interest and motivation) [3]. Definitions and models of health literacy have emerged that are more complex and comprehensive, including other elements than functional skills (literacy and numeracy), knowledge, cognitive abilities, and information processing. Especially behavioural, affective, and conative attributes have earned their place in some of the health literacy concepts and models. Furthermore, there is more attention for the context and domain in which people have to be health literate.

The development of health literacy measurement instruments, however, has not followed the movement toward a more comprehensive conceptualization of health literacy. Many of the questionnaires and tests focus on functional reading and numeracy skills (e.g., Rapid Estimate of Adult Literacy in Medicine (REALM), Test of Functional Health Literacy in Adults (TOFHLA), Set of Brief Screening Questions (SBSQ), Newest Vital Sign (NVS)) or predominantly on cognitive aspects (Short Assessment of Health Literacy (SAHL), Health Literacy Survey-Europe (HLS-EU)). There are only two instruments that employ a broader perspective: the Functional, Communicative, and Critical Health Literacy Scale (FCCHL), which measures functional, communicative, and critical health literacy [4], and the Health Literacy Questionnaire (HLQ), which includes nine different constructs including behavioural, affective, and conative elements [5]. Apart from the ‘official’ health literacy instruments, the Patient Activation Measure (PAM) also measures knowledge, skills, and self-confidence in dealing with one’s own health, (chronic) illness, and healthcare [6]. The PAM is focused on the active role of individuals and discerns four stages: (1) believing that one’s own (patient) role is important; (2) having the confidence and knowledge necessary to take action; (3) actually taking action to maintain and improve one’s health; and (4) staying on course even under stress [6]. Even though there has been debate whether patient activation and health literacy cover the same concept [7,8,9,10], with a broader definition of health literacy, there is definitely overlap, especially in the non-cognitive domain. While it is true that the PAM measures different attributes and competencies compared to most traditional health literacy instruments that focus on functional skills and/or information processing, with a broader conceptualization of health literacy, the elements of the PAM become highly relevant and of added value as well.

As with the measurement instruments, many health literacy interventions have a limited focus on reading/numeracy skills, knowledge and (cognitive) understanding of health-related issues [11,12,13,14]. This is reflected in the measurement instruments they use and in the goals that are set for the intervention. In general, these interventions improve the understanding of health information [11,12]. Effects of health literacy interventions on other outcomes are mixed or limited [11,12]. This might be because of their narrow approach. In a recent international review, the conclusion was drawn that what discriminates more promising from less promising health literacy interventions is (a) whether they tailor their efforts to the needs of the patients or groups with inadequate health literacy—patient or citizen involvement in the process of intervention development is therefore crucial—and (b) whether they address interactive and/or critical skills and competencies (and not knowledge only) [13]. If the goal of the intervention is the capacity to better self-manage, or a more active contribution in the provider-patient encounter, literacy, knowledge, and understanding of information obviously will not be enough. In traditional psychosocial models, attitudes, perceived norms and perceived behavioural control determine the intention to display certain behaviour, whereas skills and abilities further determine whether the behaviour will actually occur [15]. Both intention and skills are thus major drivers of behavioural change. Recently, in the Netherlands, a report was published on the determinants of general life skills and self-reliance of people in the domains of health, finance and work [16]. On the basis of theory and empirical evidence, a behavioural model was proposed in which, on the one hand, cognitive elements were distinguished (‘the capacity to think’), and on the other hand, non-cognitive elements (‘the capacity to act’) were distinguished. With the capacity to act, the authors refer to skills, such as goal-setting, making a plan, taking action, persevering and being able to deal with temptations and adverse events or stress. Part of these skills are determined by one’s personality structure, but other aspects are learnable. The authors demonstrated the importance of both types of capacities in order to be in control and self-reliant. Since the non-cognitive aspects are often lacking in health literacy interventions, their mixed or limited effects on behaviour are not surprising.

In this study, we will look at the impact of both the cognitive and the non-cognitive aspects of health literacy and investigate their respective effects on specific healthcare behaviours and experiences (healthcare use, experiences with patient-centered care, shared decision making, and self-management). Since an active role is especially relevant for people with a chronic condition, we focus on this specific group. Our main research questions will be:

(1) How are cognitive and non-cognitive aspects of health literacy distributed in a sample of Dutch chronically ill patients and how does this compare to the general Dutch population?
(2) How are these types of attributes (cognitive vs. non-cognitive) related to patients’ healthcare use, experiences with patient-centred care, shared decision making, and self-management?
(3) Which type of attributes (cognitive or non-cognitive) has most impact on these outcomes?

We expect that respondents who score high on both the cognitive and the non-cognitive aspects of health literacy will have the most favourable outcomes (less healthcare use, more positive experiences with patient-centred care, shared decision making, and self-management) but that the capacity to act will have a higher impact on outcomes in general than cognitive skills only.

## 2. Materials and Methods

### 2.1. Design and Data Collection

In 2017, the Dutch version of the HLS-EU questionnaire (16 items) as a measure for the functional/cognitive aspects of health literacy (‘the capacity to think’) and the Dutch Patient Activation Measure (PAM) (13 items) as a measure for ‘the capacity to act’ were included in a survey of the National Panel of the Chronically Ill and Disabled (NPCD) [17].

For this study data were used from a questionnaire sent in October 2017 to a sample of 2606 NPCD panel members who had at least one chronic disease diagnosed by a medical practitioner, and 2129 patients (81.7%) returned the questionnaire. As a reference, similar data—collected in the same period with the same instruments—from a sample of the Dutch Health Care Consumer Panel were used. This panel consists of almost 12,000 people aged 18 years and older [18]. The sample used for this study consisted of 668 people and was representative for the Dutch population regarding age and gender. Data in both panel studies are collected using online and/or paper and pen surveys.

Box 1 **National Panel of the Chronically Ill and Disabled (NPCD).**

The NPCD is a nationwide prospective panel-study on the consequences of chronic illness in the Netherlands. Panel members are recruited from the patient files of general practices (national random samples of general practices drawn from the Netherlands registration of General Practices. Patients are selected according to the following criteria: a diagnosis of a somatic chronic disease by a certified medical practitioner, an age > 15 years, being non institutionalized, being aware of the diagnosis, not being terminally ill (life expectancy > 6 months according to the General Practitioner (GP)), being mentally able to participate, and a sufficient mastery of the Dutch language. Patients who meet the selection criteria are invited by their GP to participate in the panel and asked to participate for a period of four years at a maximum. Patients who agree to participate fill in self-report questionnaires twice a year. NPCD is registered with the Dutch Data Protection Authority; all data were collected and handled according to the privacy protection guidelines of the Authority.

### 2.2. Materials

For this study, the HLS-EU 16 was used. The HLS-EU questionnaire is focused on cognitive aspects of health literacy and distinguishes four types of competences: the ability to access, understand, appraise, and apply health information [2]. In the 16-item version, 13 items refer to the first three types of competences (e.g., how easy/difficult would you say it is to (a) find information on treatments of illnesses that concern you; (b) understand what your doctor says to you; and (c) judge if the information on health risks in the media is reliable?). The three aspects that cover the application of information are mainly focused on decision making which is also a cognitive process: how easy/difficult would you say it is to (a) use information the doctor gives you to make decisions about your illness; (b) follow instructions from your doctor or pharmacist; and (c) decide how you can protect yourself from illness based on information in the media?). We used the version with five answering categories: a four-point Likert scale (very easy–very difficult) and a ‘don’t know’ option. This last category was recoded as missing. Respondents had to answer at least 14 of the 16 items in order to be included in the analysis. Subsequently the answering categories were dichotomized (easy vs. difficult) and a sum score was calculated. For respondents with 14 or 15 answers, a weighted sum score was computed by dividing the total score by the number of answered items. The final sum score varied between 0 and 16. We used the following cut off points: 0–8 is inadequate health literacy, 9–12 is limited, and 13–16 is sufficient health literacy. For more information on the HLS-EU 16 and the method of data management we used see Vandenbosch et al. [19]. In this study, we divided the respondents in two groups: inadequate/limited health literacy (HL low) and sufficient health literacy (HL high). The reason to do so is because both the people with inadequate and with limited health literacy encounter problems in healthcare and therefore require extra attention. Reliability of the HLS-EU in this study was good: Cronbach’s α was 0.87.

The PAM focuses on people’s self-perceived ability to deal with one’s own health, (chronic) illness and healthcare. The 13 items are (1) When all is said and done, I am the person who is responsible for taking care of my health; (2) Taking an active role in my own healthcare is the most important thing that affects my health; (3) I am confident I can help prevent or reduce problems associated with my health; (4) I know what each of my prescribed medications do; (5) I am confident that I can tell whether I need to go to the doctor or whether I can take care of a health problem myself; (6) I am confident that I can tell a doctor concerns I have even when he or she does not ask; (7) I am confident that I can follow through on medical treatments I may need to do at home; (8) I understand my health problems and what causes them; (9) I know what treatments are available for my health problems; (10) I have been able to maintain (keep up with) lifestyle changes, like eating right or exercising; (11) I know how to prevent problems with my health; (12) I am confident I can figure out solutions when new problems arise with my health; (13) I am confident that I can maintain lifestyle changes, like eating right and exercising, even during times of stress. It consists of 13 items, which have five answering options: a four-point Likert scale (disagree strongly–agree strongly) and a ‘not applicable’ option. For calculating patients’ activation scores, we followed the guidelines of Insignia Health [20]. According to these instructions, participants who answered less than seven questions or answered all items with ‘disagree strongly’ or ‘agree strongly’ were excluded. The mean score was calculated leaving out items that were deemed not applicable by the respondents and then transformed into a standardized activation score ranging from 0 to 100. Higher scores indicate that patients are more activated. On the basis of their mean score, patients were assigned to one of the four groups described above. The cut-off points of these groups are described in the manual of Insignia Health [21]. For this study, the two lowest groups (1 and 2) and the two highest groups (3 and 4) are taken together (respective PAM low and PAM high). On the basis of a combination of the scores on the HLS-EU 16 and the PAM, four groups were distinguished (1) HL low/PAM low; (2) HL high/PAM low; (3) HL low/PAM high; (4) HL high/PAM high. Reliability of the PAM in this study was good: Cronbach’s α was 0.78.

Outcome measures with regard to healthcare behaviours and experiences were: number of visits to GP, questions concerning patient-centered care, shared-decision making and self-management. Items to measure the latter three came from the Person-Centred Coordinated Care Experiences Questionnaire (P3C_EQ) [21]. Age, gender, education level (low i.e., no, primary school, or vocational training, middle: secondary or vocational education or high: professional higher education or university), ethnicity (Dutch, western immigrant, non-western immigrant), and self-reported health (VAS scale from the EQ 5D [22]) were included as background variables.

### 2.3. Statistical Analyses

Descriptive statistics were conducted to provide information on the characteristics of the total study sample *t*-tests ANOVA’s were used to test differences between the four groups in main outcome measures. We performed stepwise multiple regression analyses to establish which aspects of health literacy (cognitive or non-cognitive) has most impact on specific outcomes. In step 1, demographic variables were included, in step 2, the HLS-EU score (cognitive aspects) only, in step 3, the PAM score (non-cognitive aspects) only, and in step 4, both the HLS-EU and the PAM scores.

## 3. Results

### 3.1. Participants

Of the NPCD sample (*n* = 2.129), 1416 (67%) had a valid score on the HLS-EU questionnaire, and 1956 (91.9%) had a valid score on the PAM. For this study, we further selected those respondents that had a valid score on both the HLS-EU and the PAM, resulting in a sample of 1341 people with at least one chronic disease from NPCD. In Table 1, the NPCD sample is described.

In Table 1, the distribution of cognitive and non-cognitive aspects of health literacy is also presented. Health literacy as measured by the HLS-EU represents the functional/cognitive aspects of health literacy (‘the capacity to think’), whereas the PAM scores represent the non-cognitive aspects (‘the capacity to act’). Among people with a chronic disease (*n* = 1.341), 39.9% had inadequate or limited HL levels, and 36.9% had a rather low activation score, i.e., activation level 1 or 2. People in these levels often lack the motivation, knowledge, and skills to take care for their own health and well-being and therefore may need support for prevention and (self-)management. In the reference sample of the Dutch Health Care Consumer Panel, 36.4% had inadequate or limited HL levels [23], and in total, 31.6% had an activation score in level 1 (14.3%) or 2 (17.3%) (M = 63.7 (SD = 16.1)). Both the HL and the activation scores in the general population were somewhat higher, presumably as a result of differences in age and education level. Participants in the NPCD are generally older and lower educated compared to the participants in the Health Care Consumer Panel.

### 3.2. Relation of Cognitive and Non-Cognitive Aspects and Healthcare Use

One of our aims is to investigate the relationship between cognitive and non-cognitive aspects of health literacy on one hand and healthcare use on the other hand. To this purpose, we selected the sample of people with a chronic disease and divided them into four groups (see Table 2):

- Group 1: HL low, PAM low
- Group 2: HL high, PAM low
- Group 3: HL low, PAM high
- Group 4: HL high, PAM high

Related to healthcare use, there were significant differences between the four groups regarding their visits to general practitioners (% patients who had contact and number of contacts), primary care practice nurses (number of contacts), medical specialists (% patients who had contact), and use of informal care (Table 3). There were no significant differences with respect to the percentage of patients who visited the practice nurse or in the number of contacts with a medical specialist.

Looking at the relative importance of cognitive aspects versus non-cognitive aspects of health literacy, it is clear that group 1, which scores low on both, visits the GP significantly more often than patients from the other groups, and a much larger percentage of this group (differences with other groups between 5.9% and 16.1%) visits a medical specialist as well. Furthermore, the use of informal care is much larger in this group (differences with other groups between 10.9% and 18.5%). Group 4, which scores high on both, uses less care, i.e., visits the GP and the practice nurse less often, fewer people of this group visit a specialist, and there is less use of informal care. Patients in group 2 and 3 hold an intermediate position between group 1 and 4. In general, they do no not differ significantly from each other, except with respect to the use of informal care, which is much higher in the group with low PAM scores.

### 3.3. Relation of Cognitive and Non-Cognitive Aspects and Patient-Centered Care and Self-Management

Furthermore, we wanted to examine the relationship of cognitive and non-cognitive aspects of health literacy with experiences of patient-centered care and self-management. In Table 4, the results of these analyses are shown. The groups differ significantly on all outcomes.

With respect to the relative importance of cognitive versus non-cognitive aspects, it is clear that group 1 (which scores low on both) reports significally less positive experiences, whereas group 4 (which scores high on both) reports significally better outcomes on all items compared to each of the other groups. Though group 2 and 3, again, hold an intermediate position between group 1 and 4, there is a slight trend that group 3 is more positive compared to group 2, especially on the items ‘Patient feels that professional is interested his person as a whole’ and ‘Patient feels able to self-manage’. This suggests that for these items, the non-cognitive aspects of health literacy are more important compared to the cognitive aspects.

To further determine the impact of cognitive versus non-cognitive aspects of health literacy, we performed multiple regression analyses on the two outcome measures that both require an active role of the patient regarding their health and self-care. These two items were (a) shared-decision making—the extent to which the patient feels himself involved in decisions about their care (Table 5)—and (b) self-management—the extent to which the patient feels able to self-manage (Table 6).

In this regression analysis, the total amount of explained variance in step 4 is 0.11, and the cognitive aspects (HLS-EU) are of equal importance as the non-cognitive aspects (PAM): both have a value of 0.19. As is shown in model 2 and 3, the relative contribution of the HLS-EU and the PAM separate was similar. Furthermore, the educational level is an important factor throughout all three models.

With respect to the perceived ability to self-manage, we performed a second regression analysis.

The total amount of explained variance in step 4 was 0.31. Both the cognitive (HLS-EU) and the non-cognitive aspects (PAM) have a significant contribution in explaining the variance regarding the perceived ability to self-manage. The non-cognitive aspects (PAM), however, are contributing more to the R^2^ than the cognitive aspects (HLS-EU), 0.31 and 0.11, respectively. Adding the PAM in model 3 led to a 0.30 change of the explained variance. Also, the perceived general health and, to a lesser extent, educational level and the severity of the disability are important factors in all three models.

## 4. Discussion

In this study, we have investigated the impact of both the cognitive and the non-cognitive aspects of health literacy on specific healthcare behaviours and experiences (healthcare use, experiences with patient-centred care, shared decision making, and self-management).

In general, four out of 10 Dutch men and women with a chronic disease have inadequate or limited functional/cognitive health literacy skills (compared to 36% of the general Dutch population), and 37% have a low patient activation score (compared to 32% in the Dutch population). This implies that either the capacity to think or the capacity to act pose problems for a vast minority of chronically ill people and the general population alike. When we combine the scores on both types of competencies, 23% of the people with a chronic illness score low on both (group 1), whereas almost half (46%) demonstrated adequate levels in both respects (group 4). Of the intermediate groups, 14% scored high on the cognitive aspects but had low activation scores (group 2), while for 17% this was the other way around (group 3).

The main differences regarding healthcare use were found between group 1 (who scored low on both aspects of health literacy) and the other three groups. People from this group visit the GP significantly more often, and a much larger percentage of this group visits a medical specialist as well. Furthermore, the use of informal care is much larger in this group. Group 4, as expected, uses the least professional and informal care. Patients in group 2 and 3 hold an intermediate position between group 1 and 4. In general, they do not differ significantly from each other, except with respect to the use of informal care. Obviously, people who lack the capacity to act need more help and support with their health issues and self-care from their family, friends and peers than people who lack functional and cognitive skills. Hibbard et al. have earlier suggested that activation could help to compensate for numeracy and literacy skill deficits [24]. But otherwise, both functional/cognitive and non-cognitive aspects seem to be equally important determinants of healthcare use. This finding differs from the study of van der Heide et al. [25] who demonstrated that only functional skills (compared to communicative and critical health literacy skills) were associated with more visits to the GP.

With regard to patient-centred care group 1 reports significantly less positive experiences. Group 4, as expected, reports the best experiences compared to each of the other groups. Group 2 and 3, again, hold an intermediate position, but now on some aspects group 3 is slightly more positive about the care than group 2. This suggests that for some aspects of patient-centred care, non-cognitive aspects of health literacy could be more important compared to cognitive aspects. Especially for the behaviours that require an active role of the patient, the capacity to act may be relatively more important than the capacity to think. Regression analyses confirmed this for the extent to which patients feel able to self-manage, but for the extent to which patient feels involved in decisions about care (shared decision making), both the cognitive and the non-cognitive aspects proved equally important.

We can conclude from our study that both the cognitive and the non-cognitive aspects of health literacy are important and that they enhance each other. In general, patients who score low on both have the least favourable outcomes (more healthcare use, less positive experiences with patient-centred care, shared decision making, and self-management). For patients who have adequate competency levels in both respects, the outcomes are the best. Our hypothesis that the capacity to act would generally have a higher impact on outcomes compared to cognitive skills only holds for the extent to which people feel able to self-manage. Neither on healthcare use nor on other experiences with patient-centred care the non-cognitive aspects have a significant higher impact. This is an interesting finding, since in earlier studies where (functional) health literacy and patient activation were compared, patient activation was a stronger predictor with respect to outcomes such as health information seeking and use [10,24], provider choice [26], and self-management [7]. This confirms the idea that the non-cognitive aspects that are measured by the PAM (knowledge, motivation, self-confidence) are predominantly important for outcomes that require an active role such as seeking and use of health information, choice of a healthcare provider, and self-care. For those behaviours, finding and understanding of health information is not enough.

The main strength of this study is that it contributes to the further conceptualization of health literacy by establishing the influence of specific aspects and competencies on different outcomes. Since both functional/cognitive and non-cognitive aspects of health literacy prove to be important, this study underscores the importance of including non-cognitive competencies in both health literacy measurement instruments and health interventions (e.g., through enhancing motivation, personal goal setting, and skills training). Hibbard and Mahoney describe the importance of taking small, realistic, steps in interventions that aim at activating more passive patients [27]. Realizing small successes can start an upward cycle toward positive affect and self-perception, just as failure produces the opposite. People with limited health literacy tend to be passive in the medical encounter and less effective self-managers. Interventions with more focus on the ‘capacity to act’ and tailored to the individual’s health literacy and activation level will likely lead to more effective behaviour regarding health and healthcare.

A limitation of this study is that our sample is drawn from an existing panel (NPCD), in which people who are illiterate or have low literacy skills and non-Western immigrants are known to be underrepresented. The same holds for the people from our reference group. Since people with no or low literacy generally also have lower health literacy skills, this means that the percentages in our study regarding low/high health literacy are more positive than in reality. Especially group 1 will be larger than we found in this study, which is concerning given the fact that their outcomes were least favourable. Immigrants who are not able to speak or read the local language are faced with additional challenges.

Future research should use a more comprehensive conceptualization of health literacy in its design and develop and use health literacy measurement instruments that represent the multiple aspects of health literacy. A unilateral focus on functional (reading) and cognitive (understanding) competencies will neither lead to further insight into the process of how health literacy affects specific outcomes nor will it effectively guide the development of tailored interventions. It is important to get a better insight in the exact nature of the cognitive and behavioural aspects of health literacy and how they interrelate. In research as well as in development of interventions, people with low health literacy should be actively involved. That will entail new and different ways of collaboration and research. One consequence will be a lesser focus on quantitative, survey research and more attention for qualitative methods, e.g., interviews, focus groups and research material with visual cues.

## 5. Conclusions

Both the cognitive and the non-cognitive aspects of health literacy are important, and they enhance each other. Patients who score low on both use more healthcare and have less positive experiences with patient-centered care, shared decision making, and self-management. Patients who have adequate competency levels in both respects also have the best outcomes. Our hypothesis that the capacity to act would generally have a higher impact on outcomes compared to cognitive skills only holds for the extent to which people feel able to self-manage.

## Figures and Tables

**Table 1:** Characteristics of the samples.

Characteristics	People with Chronic Disease (*n* = 1.341)	
	*n*	%
Female	736	54.9
Age		
≤39 years	70	5.2
40–64 years	534	39.8
65–74 years	434	32.4
≥75 years	303	22.6
Educational level		
Low	315	24.2
Middle	585	45.1
High	399	30.7
Ethnicity		
Dutch	1228	91.9
Western immigrant	95	7.1
Non-western immigrant	13	1
Disabilities		
None/light	768	60
Moderate/severe	411	40
Number of chronic conditions		
1	483	36
2	405	30.2
3	262	19.5
≥4	191	14.2
Self-rated health (0–100)	*n*	M (Sd)
	1.318	71.2 (17.3)
Health literacy	*n*	%
Inadequate	180	13.4
Limited	355	26.5
Sufficient	806	60.1
Activation level		
1	218	16.3
2	278	20.7
3	399	29.8
4	446	33.3
Activation score (0–100)	*n*	M (Sd)
	1.341	61.2 (15.2)

**Table 2:** Level of health literacy by patient activation level in people with a chronic disease (*n* = 1.341).

Groups	HL Low	HL High
	*n* (%)	*n* (%)
PAM low	304 (22.7%)—Group 1	192 (14.3%)—Group 2
PAM high	231 (17.2%)—Group 3	614 (45.8%)—Group 4

**Table 3:** Cognitive and non-cognitive aspects of Health Literacy in relation to healthcare use of people with a chronic disease (*n* = 1.341).

Aspects of Health Care Use	Group 1	Group 2	Group 3	Group 4	*p*
	*n* = 304	*n* = 192	*n* = 231	*n* = 614	
General practitioner (% contact during last year)	93.2	94.9	91.0	87.2	<0.01
General practitioner (number of contacts during last year)	6.65	5.26 *	4.48 *	4.35 *^†#^	<0.001
Practice nurse (% contact during last year)	61.3	62.2	57.8	58.6	ns
Practice nurse (number of contacts during last year)	3.31	3.81	3.06	2.69 *^†#^	<0.01
Medical specialist (% contact during last year)	82.4	74.5 *	76.5 *	66.3 *^†#^	<0.001
Medical specialist (number of contacts during last year)	5.04	5.45	4.50	4.36	ns
Use of informal care (%)	45.7	34.8 *	27.2 *^+^	29.8 *^†#^	<0.001

* significant difference with group 1 (HL low/PAM low); ^†^ significant difference from group 2 (HL high/PAM low); ^#^ significant difference from group 3 (HL low/PAM high).

**Table 4:** Cognitive and non-cognitive aspects of Health Literacy in relation to experiences with patient-centered care and self-management among chronic disease patients (*n* = 1.341).

Extent to Which Patient…	Group 1	Group 2	Group 3	Group 4	*p*
	*n* = 304	*n* = 192	*n* = 231	*n* = 614	
Experiences patient-centered healthcare (1–4) ^1^	2.14	2.28	2.28	2.55 *^†#^	<0.001
Feels himself involved in decisions about his care (1–4)	2.74	2.97 *	3.00 *	3.31 *^†#^	<0.001
Feels that the professional is interested in him as a whole person (not only the illness) (1–4)	2.42	2.59 *	2.65 *	2.99 *^†#^	<0.001
Feels that he gets enough support in caring for his health (1–4)	2.33	2.42	2.41	2.57 *^#^	<0.001
Gets useful and timely information (1–4)	2.91	3.19 *	3.16 *	3.40 *^†#^	<0.001
Feels able to self-manage (1–4)	2.71	2.91 *	3.16 *	3.37 *^†#^	<0.001

^1^ scores range from 1 to 4 with higher scores being more positive; * significant difference with group 1 (HL low/PAM low); ^†^ significant difference from group 2 (HL high/PAM low); ^#^ significant difference from group 3 (HL low/PAM high).

**Table 5:** Extent to which patient feels involved in decisions about care (score 1–4)—stepwise multiple regression.

Variables	Model 1	Model 2	Model 3	Model 4
Constant	14.06 ***	9.38 ***	10.11 ***	7.79 ***
Female	0.01	0.01	0.3	0.02
Age	−0.9 ***	−0.05	−0.7 **	−0.05
Educational level	0.12 ***	0.10 ***	0.09 ***	0.08 ***
Immigrant	0.02	0.02	0.02	0.02
Moderate/severe disabilities	0.02	0.05	0.04	0.06
Perceived general health	0.06 *	0.01	−0.01	−0.03
HLS-EU score	−	0.25 ***	−	0.19 ***
PAM score	−	−	0.25 ***	0.19 ***
R^2^	0.03 ***	0.09 ***	0.08 ***	0.11 ***
Change in R^2^		0.06	0.05	0.08

Model 1: Background characteristics; Model 2: Background characteristics + health literacy (HLSEU-16 score); Model 3: Background characteristics + patient activation (PAM score); Model 4: Background characteristics + health literacy (HLSEU-16 score) + patient activation (PAM score); Dash indicates not applicable; * *p* < 0.05; ** *p* < 0.01; *** *p* < 0.001.

**Table 6:** Extent to which patient feels able to self-manage (score 1–4)—stepwise multiple regression.

Variables	Model 1	Model 2	Model 3	Model 4
Constant	15.44 ***	10.99 ***	10.23 ***	8.47 ***
Female	0.04	0.04	0.07 **	0.06 **
Age	−0.7 **	−0.04	−0.05	−0.03
Educational level	0.11 ***	0.09 ***	0.06 **	0.06 *
Immigrant	−0.01	−0.02	−0.01	−0.01
Moderate/severe disabilities	−0.10 ***	−0.08 ***	−0.07 **	−0.07 *
Perceived general health	0.35 ***	0.30 ***	0.25 ***	−0.24 ***
HLS-EU score	−	0.21 ***	−	0.11 ***
PAM score	−	−	0.35 ***	0.31 ***
R^2^	0.19 ***	0.23 ***	0.30 ***	0.31 ***
Change in R^2^		0.04	0.11	0.12

Model 1: Background characteristics; Model 2: Background characteristics + health literacy (HLSEU-16 score); Model 3: Background characteristics + patient activation (PAM score); Model 4: Background characteristics + health literacy (HLSEU-16 score) + patient activation (PAM score); Dash indicates not applicable.; * *p* < 0.05; ** *p* < 0.01; *** *p* < 0.001.
